# Interpersonal and individual effects of an app-based Christian and Islamic heart meditation intervention in healthy adults: protocol of a stratified randomised controlled trial

**DOI:** 10.1186/s40359-024-02022-y

**Published:** 2024-09-27

**Authors:** Chung Fei Ng, Miguel Farias, Inti A. Brazil

**Affiliations:** 1 Brain, Belief, & Behaviour Lab, Centre for Trust, Peace, and Social Relations, Coventry University, Coventry, England; 2 Radboud University, Donders Institute for Brain, Cognition and Behaviour, Nijmegen, Netherlands

**Keywords:** Christian spiritual meditation, Islamic spiritual meditation, Heart-centred spirituality, Mindfulness, Prosocial behaviour, Empathy, Forgiveness, Pain, Attention, Stress, Well-being

## Abstract

**Background:**

The academic development and widespread adoption of meditation practices for well-being and therapy have predominantly focused on secularised adaptations of Buddhist and Hindu techniques. This study aims to expand the field by investigating Christian and Islamic meditation that emphasize the spiritual significance of the heart through elements of visualisation and recitation. It compares the effects of spiritual heart-centred meditation with mindfulness meditation and a waitlist control, focusing on dimensions of social functioning, psychophysiology, cognition, and mental health.

**Method:**

This study employs a stratified 3-arm randomised controlled method with mixed-method repeated measures across three assessment time points: before intervention (T1), after an 8-week intervention (T2), and at a 3-month follow up (T3). The three conditions include spiritual meditation (either Christian or Islamic), mindfulness meditation (Mindfulness-Based Stress Reduction – MBSR), and a waitlist. Participants will be stratified into Christian and Muslim samples and randomly allocated to the spiritual meditation, MBSR, or waitlist control conditions. Importantly, participants assigned to the spiritual meditation condition will be matched to the spiritual meditation program corresponding to their religion. The intervention will be administered through a mobile phone app with daily 20-minute guided meditation sessions for eight weeks. Primary outcomes pertain to the domain of interpersonal functioning, focusing on prosociality, forgiveness, empathy, and perspective taking. Secondary outcomes include physiology: pain tolerance, pain intensity, stress reactivity assessed via heart rate (HR) and heart rate variability (HRV), psychophysiological reactivity associated with a forgiveness task as measured through HR and HRV, attention (alerting, orienting, and executive attention networks), and mental health (stress, depression, anxiety, subjective well-being, positive and negative affect).

**Discussion:**

This trial aims to test the effects of an app-based Christian and Islamic meditation, compared to secular mindfulness and a waitlist, using a randomised controlled trial. If the results yield positive outcomes, this study will support the efficacy of these contemplations, offering practitioners a way to enhance their well-being within their religious framework.

**Trial registration:**

ClinicalTrials.gov, NCT06136676. Registered on 18 November 2023.

https://clinicaltrials.gov/study/NCT06136676.

## Background and rationale

Contemplative practices have gained significant popularity since the 1970s [1, 2]. Mindfulness meditation has found a place among therapeutic settings, where practitioners employ it as a strategy to promote wellbeing [3, 4]. Derived from Buddhist and Hindu traditions, mindfulness and Transcendental Meditation are, by far, the most widely researched techniques of contemplative practices [1], with evidence suggesting that its practice can help improve mental health [5, 6], attention [7, 8], and pain management [1, 9]. However, the field of contemplative studies has overwhelmingly used secularised adaptations of meditation practices [1]. Recent criticisms of the present ‘monoculture of mindfulness’ in contemplative science remind us of the extraordinary diversity of such practices across religious traditions, which remains largely unstudied [1, 10, 11]. 

While the focus of therapeutic meditation techniques has been on the reduction of stress generated by postindustrial life [12], religious meditation strives primarily for a deep transformation of the self, where ordinary and self-oriented concerns are replaced by spiritual and prosocial oriented goals [13–15]. Although there is a large body of research that consistently shows positive outcomes of religious practice on health and well-being [16], surprisingly, there has been little interest in studying the effects of religious forms of meditation.

Another gap in the curent contemplative science literature is the study of prosocial effects. Given that these techniques were originally intended to motivate individuals to shift their self-serving interests towards spiritual and other-centred aims, the potential prosocial effects of these practices ought to be more consistently studied. Despite the growth of such studies focusing on mindfulness, loving-kindness, and compassion meditation [17–19], this research has rarely addressed traditional meditation practices, although there has been limited research into the effects of Christian prayer on prosociality tendencies [20], charitable giving [21], and the cultivation of forgiveness and empathy [22, 23]. 

Within Christian and Islamic traditions, which are the two largest Abrahamic religions worldwide, contemplative practices often seek to induce strong emotions, such as joy, love, and repentance, while engaging in recitation, visualisation, and breathing, with the aims of perfecting oneself and deepening one’s relationship with God [24–27]. Such an approach contrasts with mindfulness meditation, which seeks to attain states of calmness through techniques designed to mitigate emotional reactivity [28]. 

One important aspect that is common to Christianity and Islam is the symbolic role played by the heart [29], as evidenced by its scriptures [30–32] and contemplative practices [33–35]. There are various Christian and Islamic meditation practices that attribute particular attention to the heart. For example, in the Christian Sacred Heart of Jesus practice, individuals contemplate the sacred heart of Jesus through visualisation alongside prayer recitation. Devotion to Jesus’ heart directs an individual’s heart towards receiving and reciprocating Jesus’ love [27, 33]. In Islam, The Brilliance of Hearts meditation combines visualisation exercises with prayer recitation and strong emotional emphasis on the expression of love for God [26, 34]. Despite their particularities and varieties of expression, both traditions share elements at the technical level (heart focus), in aim (closeness to God and transforming the self), and in stimulating strong emotions (e.g. devotion, repentance, love). While there is growing evidence showing the positive effects of Christian (and, to a lesser extent, Islamic) religious practices on physical and mental health, such as reducing stress [36, 37], pain [38], and cardiovascular risk [39], the experimental evidence on the salutary effects of Christian and Islamic contemplation techniques is extremetly limited.

Given the plethora of studies on the effects of mindfulness meditation, on the one hand, and the extremely limited literature on Christian and Islamic meditation, this study proposes a mixed methods approach to understanding how these latter traditional forms of meditation might impact individuals. Hence, the study includes psychophysiology, cognition, social functioning, and mental health outcomes. In addition, because of the spiritual aims of these practices, which are expected to effect changes in how one perceives oneself and behaves towards others, the study also includes measures of religiosity. The choice of variables to study was guided both by the current state of the meditation literature (for a summary, see Oman [1]) and by the particular characteristics and aims of Christian and Islamic contemplative practices.

## Research question and hypotheses

This study addresses the effects of heart-centred meditation from the Christian and Islamic traditions, compared to secular practices, on social functioning, physiology, cognition, and mental health. The key question is whether the regular practice of Christian or Islamic heart-centred contemplation fosters positive outcomes at the interpersonal and the individual levels, as compared to a standardised mindfulness-based stress reduction intervention (MBSR) and a waitlist control condition.

To address these questions, this study will develop (a) an 8-week intervention based on heart-centered Christian and Islamic contemplative practices; (b) a stratified 3-arm randomised controlled trial (RCT) with contemplations (Christian and Islamic), an active control (MBSR) and a waitlist control. The contemplation and MBSR programs will be delivered through a mobile app.

We hypothesise that when compared to baseline, individuals undertaking the heart-centred contemplative practices will show: (1) greater prosociality, forgiveness, empathy, and perspective taking; (2) increased pain tolerance and attention, and reduced subjective ratings of pain intensity, reduced stress reactivity as measured by HR and HRV, and reduced psychophysiological reactivity associated with forgiveness as measured by HR and HRV; (3) reduced self-reported stress, depression, anxiety; (4) increased subjective wellbeing and positive affect as well as reduced negative affect. We also hypothesise that these outcomes will be greater for participants in the heart-centred contemplations compared to those in the waitlist control condition. We have no specific predictions for comparing the outcome of the religious (Christian or Islamic) *versus* MBSR interventions.

### Methods

#### Trial design

The study employs the Standard Protocol Items Recommendations for Interventional Trials (SPIRIT) [40] as the reporting guideline for this protocol and adheres to Consolidated Standards of Reporting Trials (CONSORT) [41] to ensure quality in study planning, implementation, and reporting.

**Fig. 1:**
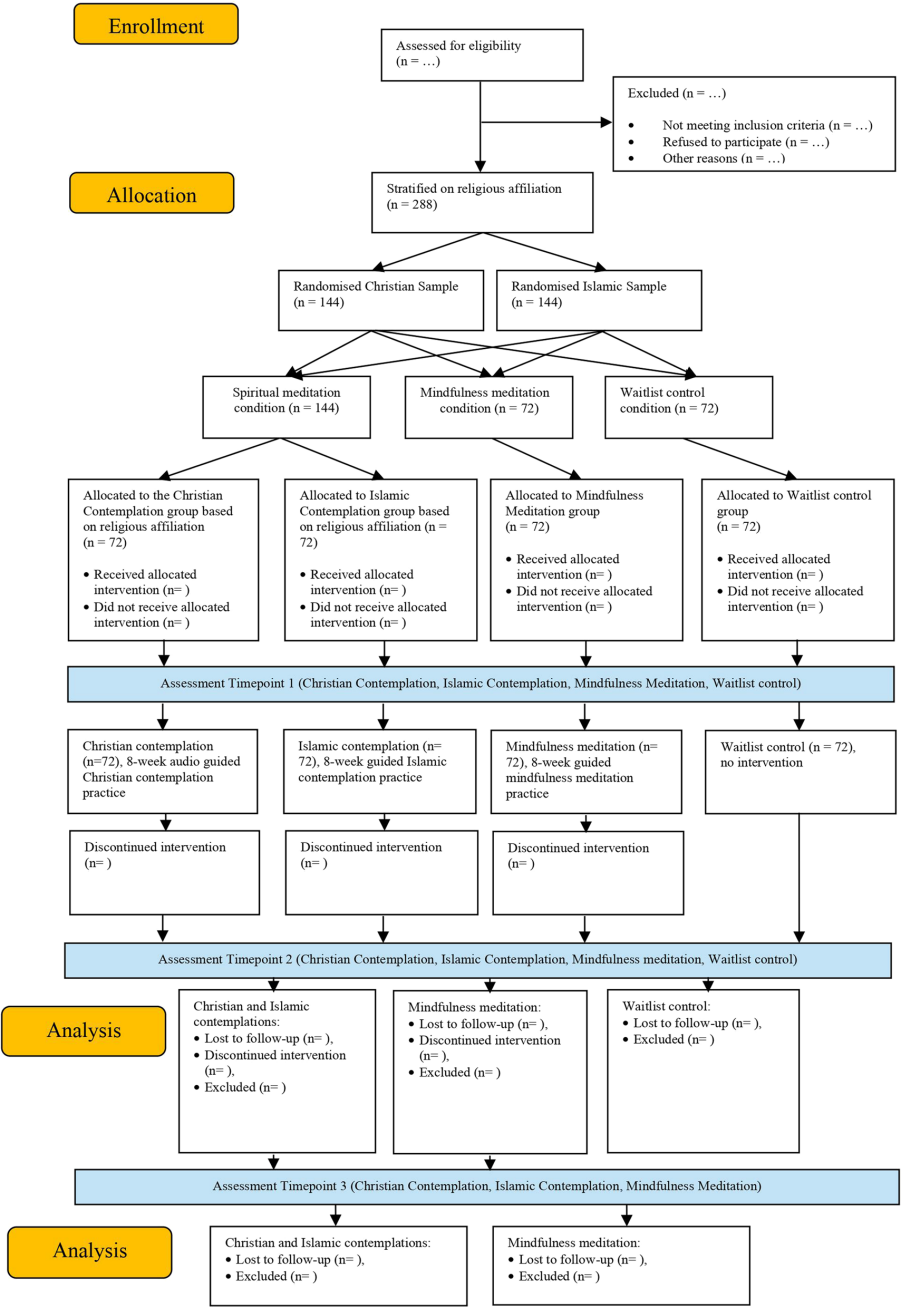
Flowchart of the study procedures

Figure 1 and Table 1 show the study procedure and the setup of the randomised control trial. This is a 3-arm stratified randomised controlled trial, with four groups distributed across two variants of the spiritual meditation condition (Christian and Islamic), MBSR, and a waitlist control condition. Participants will be first stratified into Christian and Muslim samples and then randomly allocated to one of the three conditions. Individuals assigned to the spiritual meditation condition will be further stratified into either a Christian or an Islamic contemplation subgroup within the spiritual meditation condition based on their religious affiliations.

Participants in the spiritual meditation groups and the secular mindfulness group will receive 8-week interventions, commencing with an initial face-to-face meeting, where they will practice the first session of the guided meditation course using the mobile app. Following that, they will continue with daily guided practices lasting approximately 20 min through the app. Assessment will be held at three time points including pre-intervention (T1, baseline), after the 8-week intervention (T2), and three months after the intervention (T3). Participants in the waitlist control group will only attend the assessments at T1 and T2, as due to ethical reasons they will be given the option to start using the Christian or Islamic meditation App after testing at T2.

**Table 1:**
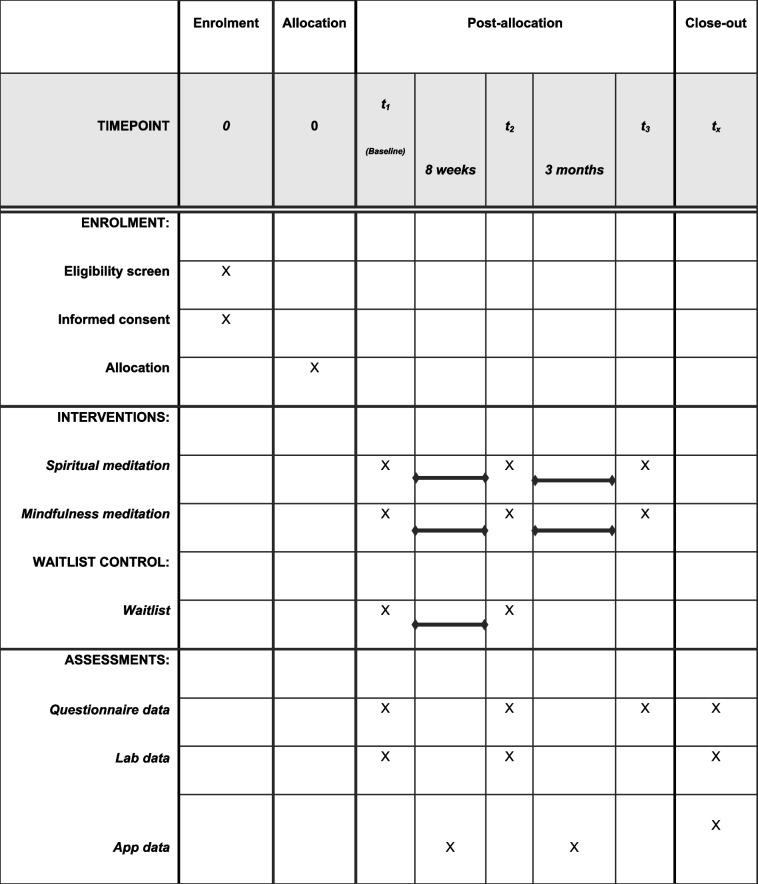
Schedule of enrolment, interventions, and assessments

## Setting

The pilot study took place in the lab at Coventry University, in England. The main trial will be conducted at two university Colleges in the state of Kerala, India, where there are large Christian and Muslim populations who are proficient in English. Two designated rooms at each College will be used as laboratories for data collection.

## Participants

### Power analysis

This study will recruit non-clinical samples of 144 Christian and 144 Islamic healthy adult participants. An a priori power analysis was conducted using the G*Power software (V.3.1) [42], considering four groups with mixed method repeated measures at three timepoints. The calculation was conducted based on a small effect size of *f*^*2*^ = 0.1, with an alpha value of 0.05, a desired power of β = 0.85. Effect size was set at 0.1 using a conservative approach due to the novel nature of the study to examine the effects of Christian and Islamic contemplation. Calculation was applied to determine the total sample size of 288 participants with (*n* = 72) per group, after adjusting for an estimated dropout rate of 10%.

## Inclusion and exclusion criteria

Inclusion criteria are: (1) 18 to 60 years old; (2) able to read, speak, and understand English; (3) willing to be assigned to any condition, with religious affiliation limiting allocation to either the Christian or Islamic type of spiritual meditation condition; (4) consider oneself to be a Christian or Muslim and pray at least weekly.

Exclusion criteria are: (1) currently suffering from any mental health conditions or using medication to manage mental health conditions; (2) having long-term serious physical medical problem, such as liver, brain, kidney, or other life-threatening chronic diseases; (3) having a history of a heart condition, high blood pressure, Raynaud syndrome, diabetes, or musculoskeletal condition; (4) the current use of anxiolytics or mood stabilizers [43]. 

## Recruitment, screening, and enrolment

The recruitment process started on 22 July 2024. Non-clinical samples of Christian and Islamic healthy adult participants will be recruited and enrolled from the local institutions through their internal participant pools, online adverts, distributing flyers, and collaboration with religious communities.

Potential participants will be contacted via email with detailed information in a written form and will be required to fill out a pre-screening online questionnaire. Their self-reports will be assessed through inclusion and exclusion criteria to determine their eligibility. The reason for ineligibility will be retained in accordance with CONSORT standards [41]. 

Eligible participants will receive information about the study including its objective and procedures. Subsequently, research team member will ask participants to provide informed consent for study enrolment. All enrolled participants will be invited to take part in the baseline assessment (T1) and the follow-up assessment (T2) with measures of social functioning, physiology, and cognition. A set of online measures concerning mental health, religiosity, socioeconomic status, demographics, social value orientation, and meditation social desirability will be taken at home before T1. Additionally, participants in the religious contemplation groups and MBSR will attend an online 3-month follow up assessment. All participants will be compensated with an INR 2,000 as compensation for their time.

### Randomisation and allocation

First, participants will be stratified into Christian and Islamic samples. They will then be randomly assigned to the spiritual meditation (either Christian or Islamic meditation, with religious affiliation restricting allocation to either the Christian or Islamic type of contemplation), MBSR, or waitlist control, utilizing a random number generator produced by a computer and allocated to one of the four groups. To ensure allocation concealment, the allocation process will be carried out by a researcher who is independent of the trial team.

### Blinding

Neither the participants nor assessors can be completely blinded given that it is not possible to create a pseudo-Christian or Islamic meditation condition. To reduce potential biases, participants will be blind to the specific study hypotheses, and will only have access to the meditation app version exclusively for their assigned groups. Additionally, the researcher responsible for the randomisation and allocation process will not participate in the recruitment, assessment, or data analysis. Considering that the trial will not involve any harmful procedure, and participants will be informed about the three arms during the recruitment stage, procedure for unblinding is not required for this trial.

### Study interventions

The spiritual meditation interventions and MBSR will have the same structure and time frame. All interventions will be delivered through an initial face-to-face meeting, where participants will initiate the first session of the guided meditation course using the mobile app. Subsequently, they will continue with daily guided practices lasting approximately 20 min through the app over eight weeks. The initial meeting will start at the end of the baseline assessment (T1). Participants will only be able to access the app intervention that they are allocated to. Participants in the waitlist control group will not receive any types of intervention and will not attend the T3 assessment, but they will be given access to the Christian or Islamic meditation app version upon completion of the in-person T2 assessment.

### Intervention content

All interventions will be delivered through an 8-week program of daily audio-guided meditations of approximately 20-minutes. There will be a total of eight different meditations per condition, one per week, where each meditation is repeated across seven days. To ensure participant engagement in the meditation practice, the app will unlock the next session for the participant only after completing the preceding day’s session. Similarly, progression to the next week’s meditation session requires completing at least four sessions in the preceding week. The app will track participants’ completion of practice sessions and record daily responses to two brief questions about mood and meditation experience. Participants can maintain their daily religious practices during the 8-week program.

### Heart-centred spiritual meditations

The spiritual meditation programs were developed from techniques based on Christian and Islamic traditions. The primary elements of the practices consist of maintaining attention and emotional connection to God through visualisation and recitation. Concurrently these practices are expected to promote a moving away or transcendence from everyday concerns. Breathing is included as an added ingredient to help focus participants’ attention during the first part of each meditation. Although Christian and Islamic contemplations are typically not standardised within their respective contemplative traditions, for this study we had to develop specific scripts to be used by participants. These were developed by Christian and Islamic expert members of the research team, and subsequently edited by the lead researchers with the guidance of an expert in writing content for meditation Apps (see acknowledgements section).

### Mindfulness-based stress reduction

A separate MBSR program was developed by a certified instructor who has experience in working for meditation Apps. The MBSR program includes an 8-week program of daily audio-guided mindfulness practice lasting approximately 20 min, based on the manual developed by John Kabat-Zinn [44]. 

### Waitlist condition

Participants assigned to the waitlist control condition will take part in the assessments at T1 and T2 but will not participate in any of the interventions. They will be given access to either the Christian or Islamic spiritual meditation app version after they complete the assessment at T2.

### Study variables

Table 2 provides a summary of the variable domains, study variables, measures, assessment time points and the types of data collection method for each variable.

**Table 2:** Overview of the outcome measures and points of assessment

Domain	Variables	Measure	Type of data collection method	Assessment point			
				Pre-intervention (T1)	Post-intervention (T2)	At 3-month follow-up (T3)	Throughout the 8-week intervention
Primary outcome measures							
Interpersonal functioning	Prosociality	Dictator Game: number of points (0–10) that a participant is willing to allocate to another individual.	Lab	X	X		
	Prosociality	Social value orientation task	Online	X	X		
	Prosociality	Charitable Giving: percentage of monetary compensation donated to a charity.	Lab		X		
	Forgiveness	Forgiveness scale assessed as part of Love thy Enemy task	Lab	X	X		
	Empathy	Empathy scale assessed as part of Love thy Enemy task	Lab	X	X		
	Perspective Taking	Interpersonal Reactivity Index (IRI)	Online	X	X	X	
Secondary Outcome measures							
Physiology	Pain Tolerance	Endurance (in seconds) of cold painful stimulation	Lab	X	X		
	Pain Intensity	0–10 Rating Scale	Lab	X	X		
	Stress Reactivity	HR	Lab	X	X		
	Stress Reactivity	HRV	Lab	X	X		
	Psychophysiological reactivity associated with forgiveness	HR assessed during forgiveness task.	Lab	X	X		
	Psychophysiological reactivity associated with forgiveness	HRV assessed during forgiveness task	Lab	X	X		
Attention	Alerting Attention network	Reaction times assessed through a 10-minute version of the Attention Network Task (CRSD-ANT)	Lab	X	X		
	Orienting Attention network	Reaction times assessed through the (CRSD-ANT)	Lab	X	X		
	Executive Attention network	Reaction times assessed through the CRSD-ANT	Lab	X	X		
Mental health	Stress	Stress subscale of the Depression, Anxiety and Stress Scale (DASS-21)	Online	X	X	X	
	Depression	Depression subscale of the DASS-21	Online	X	X	X	
	Anxiety	Anxiety subscale of the DASS-21	Online	X	X	X	
	Subjective Wellbeing	Well-Being Index (WHO-5)	Online	X	X	X	
	Positive and Negative Affect	Positive and Negative Affect Schedule (PANAS) Short Form (I-PANAS-SF)	Online	X	X	X	
	Positive and Negative Affect	Smiley Face Likert scale	Phone App				X
Other variables							
Spirituality	Spiritual experiences	16-item Daily Spiritual Experience Scale	Online	X	X	X	
	Closeness to God/Inclusion-of-God-in-the-self	Pictorial closeness to God / Inclusion-of-God-in-the-self	Online	X	X	X	
	Religiosity	5-item Duke University Religiosity Index	Online	X			
	Credibility/expectancy of the meditation practices	2-item scale	Online	X			
	Meditation adverse events	Self-report scale on meditation unpleasant events and harm	Online		X	X	
	Closeness to the offender	Pictorial Inclusions of Others in the Self scale with the Love thy Enemy task	Online	X			
Other	Age	Self-report	Online	X			
	Ethnicity	Self-report	Online	X			
	Gender identity	Self-report	Online	X			
	Sex	Self-report	Online	X			
	Employment status	Self-report	Online	X			
	Education level	Self-report	Online	X			
	Annual household income	Self-report	Online	X			
	Experiences of the App-based exercises	Self-report	Online		X	X	
	Frequency of the contemplative or mindfulness practices after the end of the intervention	Self-report	Online			X	
	Qualitative assessment (Paper diary)	Themes emerging from participant’s meditation experiences					X
	Qualitative assessment (App)	Themes emerging from participant’s daily meditative experiences					X

### Primary outcomes

The study is primarily assessing changes in interpersonal functioning, measured through prosociality, forgiveness, empathy, and perspective taking. Prosociality will be assessed at T1 and T2 via the *Dictator Game* (DG) [45], *Social Value Orientation* (SVO) [46], and *Charitable Giving* (at T2 only). Empathy and forgiveness variables will be collected via the task *Love thy Enemy* (adapted from Witvliet et al. [47]) at both T1 and T2. Perspective taking will be obtained online at T1, T2, and T3.

Dictator Game (DG) [45]: This computer-based task allocates ten game points to participants and asks them to decide how many of these points they would like to transfer to an anonymous participant. The outcome measure is the number of game points participants are willing to allocate to the anonymous individual. The game points retained by participants will have no influence on their real-life earnings or their compensation fee for taking part in the experiment. Prior studies have shown that individuals engage in prosocial activities irrespective of monetary incentives [48, 49]. 

Social Value Orientation (SVO) [46]: This online task consists of six items with which participants are asked to assign game points for themselves and an anonymous other. Participants will make their allocations by adjusting a slider to indicate their most preferred joint distribution between themselves and the other. A continuous social value orientation score will be computed from these six items based on participants’ choices. The outcome measure will be a single overall SVO score. A higher SVO score represents a higher prosocial preference. The game points kept by the participants will not affect their real-life earnings or their compensation fee for taking part in the experiment.

#### Charitable giving

Participants will be given a choice to allocate a part of their compensation fee to a charity (ranging from 0 to 100%). The prosocial outcome measure will be the percentage of the fee they are willing to allocate to a charity (Christian or Islamic, based on the participant’s religious affiliation).

Love thy enemy task (adapted from Witvliet et al. [47]): In this forgiveness task participants will focus on an enemy who has deeply hurt them; self-reported empathy and forgiveness are assessed, alongside HR and HRV to measure psychophysiological reactivity associated with forgiving the offending individual. Participants will first be asked to think of an individual who has deeply hurt them. They will then write down the initials of that person’s name, as well as when and where the specific incident happened on a piece of paper, which they will subsequently seal in an envelope (the envelop will remain sealed until T2, when participants will be asked to recall the same individual and incident). After writing down this information, participants will indicate the level of closeness they feel towards that individual (adapted from Aron et al. [50]). Once participants indicate the relationship with the person, they will proceed to the task, which consists of three phases: baseline, imagery, and recovery. In the baseline and recovery phrases (20 s for each), participants will be instructed to close their eyes and relax. In the imagery phase (40 s of duration) participants will be prompted to imagine themselves forgiving the person who hurt them. After the recovery phase, participants will rate their level of empathy towards that person (on a scale of 0–20) and the extent to which they feel they have forgiven the person during the task (on a scale of 0–20). The task will consist of four repeated trials.

Perspective Taking [51]: The 7-item Perspective Taking subscale from the Interpersonal Reactivity Index (IRI) will be used to assess participants’ tendency to take others’ points of view using a 5-point Likert scale, ranging from A (Does not describe me well) to E (Describes me very well). Higher scores indicate greater perspective-taking.

### Secondary outcomes

Secondary outcomes encompass domains of physiology, attention, and mental health. Measures pertaining to the physiological and attention domains will be obtained at T1 and T2, while those related to mental health domain will be collected across all timepoints (T1, T2 and T3).

### Physiological domain

Pain tolerance, pain intensity, and stress reactivity (HR and HRV) will be assessed using a cold pressor task (adapted from a previous spiritual intervention study [52]). Participants will immerse their non-dominant hand in the cold water at 2 °C to 3 °C. They will be instructed to endure the pain for as long as they can tolerate, up to a maximum of 4 min, with an option to remove it any time if the pain becomes unbearable. HR and HRV will be continuously measured throughout the task. After removing their hand from the cold water, participants will rate their average pain intensity experienced during the task ranging from 0 (no pain) to 10 (worst imaginable pain). A 5-minute resting state HR and HRV will be measured before the task, with another 5-minute HR and HRV measurement starting 1 min after the end of the cold pressor task. Custom computer software will record the duration (in seconds) that a participant endures painful stimulation during the cold pressor task as well as HR and HRV. The four variables assessed include (1) the duration of cold-water endurance (pain tolerance), (2) differences in HR and HRV before, during and after the task (stress reactivity), and (3) the average subjective pain intensity.

For HR and HRV measurement, participants will wear a Polar H10 heart rate sensor [53] around their chest to measure HR and interbeat intervals for quantifying the metric of root mean square of successive RR interval differences (RMSSD) for HRV. A software program has been customized to interface with the modified version of the OpenHRV application [54]. 

### Attention domain

Posner and Petersen [55] identified three distinct networks linked to different aspects of attention: one for alerting, involving right frontal and parietal regions; another for orienting with frontal and parietal lobe involvement; and a third for executive control linked to the anterior cingulate and dorsolateral prefrontal cortex. The Attention Network Task (ANT) was designed to assess the efficiency of each network through behavioural data [56]. This study uses the 10-minute version of the ANT (adapted from the attention network task) [56, 57]. 

Throughout the task, a fixation cross will be presented in the centre of the screen. Brief cue stimuli, lasting for 100ms, will appear above or below the fixation cross (spatial cue), with two cues - one above and the other below the fixation cross (double cue), at the centre of the fixation cross (centre cue), or not at all (no cue). These cues will consistently precede the target presentation. Centre and double cues provide timing information regarding when the target will appear, concerning alerting attention, while spatial cues also indicate where the upcoming target would be, involving orienting network. The target stimuli consist of five horizontally arranged arrows, positioned above or below the fixation cross, with a maximum duration of 1500ms. Participants are instructed to press the left or right arrow key on their keyboard to indicate the direction of the central arrow, regardless of the flanking conditions, with all arrows either pointing in the same direction (congruent flankers) or pointing in different direction from the central arrows (incongruent flanks). Reaction time (RT) difference across task conditions serve as outcome measures, which reflect the engagement of corresponding attention network, with a faster RT indicating a greater alerting, orienting, or executive control of attention, depending on the task type.

#### Mental health domain

This domain includes measures of stress, depression, anxiety, subjective welling, and positive and negative affect.

Stress, depression, and anxiety: These variables will be assessed using the 21-item Depression, Anxiety and Stress Scale (DASS-21) [58]. Previous research has indicated that the DASS-21 exhibits comparable levels of internal consistency across various cultures and countries [59, 60]. The scale comprises three subscales: depression, anxiety, and stress, each comprising seven items. Participants will rate how they feel over the preceding week using a 4-point Likert scale ranging from 0 to 3, where 0 indicates “Did not apply to me at all” and 3 represents “Applied to me very much or most of the time”. Total subscale scores will be computed by summing all item responses, with higher scores indicating greater symptom severity.

Subjective well-being (WHO-5) [61]: The WHO-5 comprises five items that requires participants to assess their feelings over the preceding two weeks using a 6-item Likert scale ranging from 0 to 5, where 0 represents “At no time” and 5 indicates “All of the time”. A higher score indicates a greater sense of wellbeing.

Positive and negative affect (I-PANAS-SF) [62]: This consists of a 10-item scale with higher scores indicating greater positive or negative affect.

##### Mood

A 1-item Smiley Face Likert scale within the meditation app will ask participants to assess how they are currently feeling before and after each meditation or mindfulness session. Participants select one of the five emoji faces, arranging from very sad (leftmost) to very happy (rightmost).

### Other variables

Other variables include spirituality domain related to measures of spiritual experiences, closeness to God, religiosity, demographics, socioeconomic status, and control variables.

#### Spirituality domain

##### Spiritual experiences

Spiritual experiences will be measured using the 16-item Daily Spiritual Experience Scale (DSES) [63]. Participants will rate the frequency of specific spiritual experiences using a Likert scale. While one item employs a 4-point Likert scale, with a higher score representing greater spiritual experiences, the remaining 15 items are measured on a 6-point Likert scale and are reverse-scored, with lower scores signifying greater spiritual experiences. This assessment will be conducted across all time points (T1, T2, and T3).

##### Closeness to god/inclusion-of-god-in-the-self

The measurement will employ a pictorial inclusion of God scale adapted from the Inclusions of Others in the Self scale (IOS) [50], specifically tailored to assess closeness to God. The scale comprises seven pairs of circles with varying degrees of overlap, describing the relationship between oneself and God. Greater overlap signifies a closer relationship with God, while less overlap indicates a more distant relationship. This measure will be collected across all time points (T1, T2, and T3).

##### Religiosity

It will be assessed at T1 using the 5-item Duke University Religiosity Index (DUREL) [64], measuring three primary dimensions of religious involvement: organizational religious activity (ORA) and non-organizational religious activity (NORA), and intrinsic religiosity (IR). Participants rate their involvement (ORA and NORA) on a 6-point Likert scale ranging from 1 (Never) to 6 (More than once a week) for two items, and their religious beliefs or experiences (IR) on a 5-point Likert scale ranging from 1 (Definitely not true) to 5 (Definitely true of me). Higher scores indicate a stronger religiosity.

#### Control variables

##### Credibility/expectancy of the meditation practices

A modified version of the 2-item self-report questionnaire (adapted from Creswell et al. [65]) will be used to assess participants’ belief about the relevance and the efficacy of the meditation and mindfulness interventions at T1. Higher scores indicate greater positive expectancies.

Potential adverse events (adapted from Baer et al. [66]): This questionnaire assesses unpleasant experiences and potential harm from the meditation practices and includes items with Likert. It will be used at T2 and T3.

#### Sociodemographic variables

These include age, ethnicity, gender identity, sex, employment status, education level, and annual household income.

### Other variables that may affect the outcome

#### Closeness to the offender

The measurement will employ the Inclusions of Others in the Self scale (IOS) [50], comprising seven pairs of circles with varying degrees of overlap (1, no overlap, to 7, maximal overlap) describing the relationship between oneself and the other. Participants will indicate their relationship with the one who caused them harm by selecting the pair of circles that best reflects their relationship. The assessment will be conducted at T1 with the Love thy Enemy task.

#### Experiences of the app-based exercises

Participant will be asked to provide feedback on their meditation experiences. This assessment will involve completing questionnaire containing Likert-scale items and open-text questions at T2 & T3.

#### Frequency of the meditation or mindfulness practices after the end of the intervention

Participants will complete a self-report assessment regarding the frequency of their practices following the completion of the 8-week intervention.

### Qualitative measure

Participants will be provided with a notebook and instructed to keep a diary for reflective purposes. The diary will serve to evaluate any potential adverse events associated with the meditation practices, as well as to explore the effects of the practice on participants’ daily life. Furthermore, participants will be prompted to provide feedback on their daily meditative experiences within the app following each session. This prompt aims to evaluate their experiences during meditation practices, focusing primarily on mood, emotions, and the effectiveness of the session. These measures will be assessed throughout the 8-week intervention.

## Adverse events and safety monitor

We have established an expert panel to guide the study’s design and intervention. Additionally, a dedicated study coordinator oversees the logistical and operational aspects of our research. Given that the potential for adverse events linked to meditation practices [67], we will assess this risk by including a self-report measure for adverse events. Participants will be also asked to reflect on their experiences during the practices. To address concerns, participants can consult religious experts from our advisory board regarding any potential adverse effects of the interventions. They will also be encouraged to promptly contact the lead researcher if adverse events occur, as outlined in the participant information sheet.

Participants undergoing the cold pressor task procedures may experience discomfort or pain, inherent to the procedure’s design. They will be informed that they have the option to discontinue the procedure and remove their hand from the cold water at any time. The cold pressor task is a well-established procedure, and unexpected adverse effects related to the cold pressor task procedure are unlikely to occur. Therefore, based on our risk assessment and the low anticipated risk, a formal data monitoring committee and modifications of allocated intervention are considered unnecessary for this study. It is also unlikely that participants will require post-trial care. However, the lead researcher will closely monitor and document any potential adverse events. Participants will be informed of their right to withdraw from the trial at any time without needing to provide a reason.

### Analysis plan

#### Quantitative data

The analyses will follow both intention-to-treat principle and per-protocol principle, requiring a minimum completion of four days of meditation sessions per week over the 8-week intervention period and complete all required assessments. This study will consider a result with a p-value lower than 0.05 as statistically significant. A series of mixed-design Repeated Measures Multivariate Analysis of Variance (RM-MANOVA) will be used to test the mean difference in the outcomes among conditions (Spiritual meditation, mindfulness, and waitlist control groups) across T1, T2 and T3 as well as within participants across all time points (T1, T2 and T3). The specific prosociality variable, measured by Charitable Giving task, will be only compared between groups at T2. When justifiable, missing data will be imputed using multiple imputation of chained equations [68]. 

##### Qualitative data

Thematic analysis will be employed to evaluate the data collected from participants’ diaries regarding their reflections on daily practices and meditation experiences shared in the App, following the 6-step analysis process suggested by Braun and Clare [69]. In step 1, researchers will thoroughly read the diaries and meditation experience multiple times to familiarise themselves with and understand the meaning of the content. In step 2, initial codes will be produced from the diaries and the meditation experience, then organised into meaningful groups. In step 3, codes will be organised into overarching themes. In step 4, themes will be reviewed. In step 5, each identified theme will be defined and labelled. Step 6 consists of the final analysis, selection of representative texts for the themes, and analysis write-up. Data processing and analysis will be conducted using the software NVivo [70]. 

#### Interim analyses and stopping guidelines

Given that this trial does not involve harmful procedures, stopping guidelines are not necessary, and no interim analyses are planned. However, participants can withdraw from the study or the tasks anytime without giving a reason.

## Data management and confidentiality

Each participant will be allocated a unique participant number. Raw data will be safely and securely stored on university servers that are only accessible to the research team. All identifiable data, including name and contact details, will be encrypted, and kept apart from research data. Non-digital data will be securely locked in a cabinet with access restricted to the research team.

## Dissemination plan

Anonymised data, analysis scripts and results will be shared and archived through the Donders Repository (https://data.donders.ru.nl) in accordance with open science principles after the main results of the study have been published.

### Patient and public involvement

This study aims to address a gap in contemplative sciences, where most studies narrowly focus on secularised adaptations of Buddhist and Hindu exercises. Patients will not be involved in any part of the study, including study design, recruitment, participation, or assessment.

## Discussion

Contemplative practices have drawn substantial interest over the recent decades. Among them, secular mindfulness meditation has become widely recognised and particularly popular, especially in therapeutic settings where it is frequently used to promote wellness. However, this excessive focus on a single Buddhist-based technique has led to a neglect in exploring the plethora of meditation techniques from other spiritual traditions, and their potential benefits.

To advance the field of contemplative studies and provide additional opportunities to enhance well-being, this study has developed a stratified 3-arm randomised controlled to examine the effects of Christian and Islamic spiritual meditation through a mobile app. This study will compare the effects of Christian and Islamic spiritual meditation, against mindfulness meditation and a waitlist. If the study demonstrates positive outcomes, it could enable practitioner to improve their well-being within their own religious framework.

As the interventions will be delivered online instead of in person, limitations arise in assessing whether participants fully adhere to the practice while listening to the audio instructions. For instances, they may focus on other activities, rather than on the practice. Additionally, the environment in which a participant practices may play a role in the research outcomes.

### Trial status

Study protocol version 1 (14 August 2024). Recruitment commenced on 22 July 2024. The anticipated completion date of recruitment will be 31 January 2025.

## Data Availability

No datasets were generated or analysed during the current study.

## Abbreviations

ANT: Attention Network Task
App: Application
CONSORT: Consolidated Standards of Reporting Trials
DASS-21: 21-item Depression, Anxiety and Stress Scale
DG: Dictator Game
DSES: Daily Spiritual Experience Scale
DUREL: Duke University Religiosity Index
HR: Heart Rate
HRV: Heart Rate Variability
I-PANAS-SF: International Positive and Negative Affect Schedule Short Form
IR: Intrinsic religiosity
MBSR: Mindfulness-Based Stress Reduction
NORA: Non-organizational religious activity
ORA: Organizational religious activity
RT: Reaction time
SPIRIT: Standard Protocol Items Recommendations for Interventional Trials
SVO: Social Value Orientation
T1: Timepoint 1
T2: Timepoint 2
T3: Timepoint 3
WHO-5: 5-item World Health Organization Well-Being Index
